# Systemic Sclerosis Sera Impair Angiogenic Performance of Dermal Microvascular Endothelial Cells: Therapeutic Implications of Cyclophosphamide

**DOI:** 10.1371/journal.pone.0130166

**Published:** 2015-06-15

**Authors:** Annalisa Borghini, Mirko Manetti, Francesca Nacci, Silvia Bellando-Randone, Serena Guiducci, Marco Matucci-Cerinic, Lidia Ibba-Manneschi, Elisabetta Weber

**Affiliations:** 1 Department of Molecular and Developmental Medicine, University of Siena, Siena, Italy; 2 Department of Experimental and Clinical Medicine, Section of Anatomy and Histology, University of Florence, Florence, Italy; 3 Department of Experimental and Clinical Medicine, Division of Rheumatology, Azienda Ospedaliero-Universitaria Careggi (AOUC), University of Florence, Florence, Italy; University of Bari Medical School, ITALY

## Abstract

In systemic sclerosis (SSc), dermal capillaries are progressively lost with consequent chronic tissue hypoxia insufficiently compensated by angiogenesis. Clinical studies reported that intravenous cyclophosphamide (CYC) may improve SSc-related peripheral microvascular damage. Recently, we showed that CYC treatment may normalize SSc sera-induced abnormalities in endothelial cell-matrix interactions. Our objective was to evaluate *in vitro* the effects of sera from treatment-naïve or CYC-treated SSc patients on dermal blood microvascular endothelial cell (dMVEC) angiogenesis, migration, proliferation and apoptosis. dMVECs were challenged with sera from 21 SSc patients, treatment-naïve (n = 8) or under CYC treatment (n = 13), and 8 healthy controls. Capillary morphogenesis on Geltrex matrix was significantly reduced upon challenge with sera from naïve SSc patients compared with healthy controls. When dMVECs were challenged with sera from CYC-treated SSc patients, their angiogenic capacity was comparable to that of cells treated with healthy sera. Wound healing capacity and chemotaxis in Boyden chamber were both significantly decreased in the presence either of naïve or CYC-treated SSc sera compared with healthy sera. WST-1 assay revealed that cell proliferation was significantly decreased in dMVECs challenged with sera from naïve SSc patients compared with healthy sera. Conversely, dMVEC proliferation was not impaired in the presence of sera from CYC-treated SSc patients. Accordingly, the percentage of TUNEL-positive apoptotic dMVECs was significantly higher in the presence of sera from naïve SSc patients than healthy controls, while CYC-treated SSc sera did not induce dMVEC apoptosis. Levels of the angiostatic mediators endostatin, pentraxin 3, angiostatin and matrix metalloproteinase-12 were all significantly elevated in sera from naïve SSc patients compared with sera from both healthy controls and CYC-treated SSc patients. In SSc, CYC treatment might boost angiogenesis and consequently improve peripheral microangiopathy through the normalization of the endothelial cell-matrix interactions, reduction of endothelial cell apoptosis and rebalance of dysregulated angiostatic factors.

## Introduction

Systemic sclerosis (SSc) is a chronic connective tissue disease characterized by microvascular abnormalities, production of autoantibodies and progressive fibrosis of the skin and internal organs [1,2]. Two different subsets of SSc are commonly recognized: limited cutaneous SSc (lSSc) and diffuse cutaneous SSc (dSSc), which differ in the extent of dermal fibrosis, internal organ involvement, autoantibodies, prognosis and survival [1–3]. In both forms the first symptom, that may precede of several years the onset of fibrosis, is Raynaud’s phenomenon, a reversible vasospasm of hands and feet which may lead in time to digital ulcers and even gangrene of the extremities with a major impact on patients’ quality of life [4,5]. In SSc, nailfold videocapillaroscopy highlights several microvascular abnormalities which culminate in the loss of peripheral capillary vessels leading to chronic tissue ischemia [5–7].

Tissue ischemia and hypoxia are usually the main triggers for angiogenesis through the upregulation of proangiogenic factors, which overcome angiostatic factors and initiate angiogenic sprouting from pre-existing microvessels by inducing vasodilation and activation of microvascular endothelial cells (MVECs) [8,9]. During angiogenesis, activated MVECs lose connections with each other, release proteolytic enzymes that degrade the basement membrane, migrate into the surrounding extracellular matrix, proliferate and assemble in capillary tubes. A vascular lumen is then formed and the vessel wall is eventually stabilized by the recruitment of supporting cells as pericytes and smooth muscle cells [8,9].

Despite chronic MVEC activation/damage and progressive reduction in peripheral capillary density, in SSc vascular recovery appears to be precluded by a dysregulated and insufficient angiogenic process [5,10–12]. An imbalanced expression of a wide array of circulating proangiogenic and angiostatic factors may be largely responsible for this complex scenario [5,10–16]. Moreover, an impaired response to proangiogenic stimuli and several functional defects have been reported in skin MVECs and peripheral blood-derived endothelial progenitor cells from SSc patients [10–12,17–26]. However, the question why the damaged microvessels in SSc are insufficiently replaced by new ones *via* angiogenesis or vasculogenesis is still unresolved. As a consequence, currently there still are few therapeutic options to promote effective angiogenesis and regeneration of the peripheral microcirculation [27–31].

In the present study, we evaluated whether sera from lSSc and dSSc patients may affect the angiogenic performance of human adult dermal blood MVECs (dMVECs). For this purpose, we tested the capacity of dMVECs to i) align and form capillary-like tubes *in vitro*, ii) migrate and proliferate in response to mechanical injury, and iii) migrate in response to a chemotactic stimulus. Moreover, we specifically assessed whether lSSc and dSSc sera may impair proliferation and induce apoptosis of dMVECs. Since in SSc previous studies have shown that cyclophosphamide (CYC) treatment may clinically improve microvascular damage, as assessed by nailfold videocapillaroscopy [32], and normalize aberrant endothelial cell-matrix interactions *in vitro* [33], the possible effect of sera from SSc patients treated with CYC on the angiogenic capacity, proliferation and apoptosis of dMVECs was also investigated.

## Materials and Methods

### Patients and serum samples

Serum samples were obtained from a total of 21 consecutive patients (17 women, 4 men) classified as SSc [34] and recruited from the Division of Rheumatology, University of Florence, Florence, Italy. Patients with symptoms overlapping with those of other autoimmune, rheumatic and/or connective tissue diseases were excluded from the study. Eight age-matched and sex-matched healthy individuals were used as controls. Patients were further classified in the limited SSc (lSSc; n = 13) or diffuse SSc (dSSc; n = 8) subsets [35]. All SSc patients were clinically assessed as previously described [15,33]. Thirteen patients (8 lSSc and 5 dSSc) were receiving monthly intravenous infusion of CYC (dose range, 1 to 1.5 g/m^2^ for 12 to 18 months), and the other eight patients were not taking any immunosuppressant or disease-modifying drugs. Blood was drawn from CYC-treated patients 1 month after the last infusion. Before blood sampling, all patients were washed out for 10 days from oral vasodilating drugs and for 2 months from intravenous prostanoids. Fresh venous blood samples from patients and healthy controls were allowed to clot for 30 minutes before centrifugation at 1,500 *g* for 15 minutes. Serum was collected and stored in aliquots at –80°C until used. All SSc patients and control subjects signed an informed consent form, and the study was conducted in compliance with the principles of the Declaration of Helsinki and was approved by the local institutional review board at the Azienda Ospedaliero-Universitaria Careggi (AOUC), Florence, Italy.

### Cells

Human adult dMVECs were obtained from Lonza (HMVEC-dBlAd; Lonza, Milan, Italy). These cells are ≥90% pure, express CD31, CD105, von Willebrand factor and demonstrate acetylated low density lipoprotein uptake according to the manufacturer’s certificate. Three cell lines from different donors were used in the experiments. dMVECs were cultured according to the manufacturer’s instructions in complete Endothelial Growth Medium 2 (EGM-2) supplemented with the EGM-2-MV BulletKit (Lonza) until confluent. Once at confluence, cells were trypsinized with a trypsin/ethylenediaminetetraacetic acid solution (Lonza), centrifuged, resuspended in medium with EGM-2-MV and seeded onto appropriate supports for the different assays.

### *In vitro* capillary morphogenesis assay on Geltrex

Geltrex reduced growth factor basement membrane matrix (Invitrogen, Carlsbad, California, USA) was used for the *in vitro* capillary morphogenesis assays. Culture wells (BD Falcon 96-multiwells, well surface 0.32 cm^2^; BD Biosciences, San Diego, California, USA) were coated with Geltrex (32 μl/well). Geltrex was allowed to polymerize 30 minutes at 37°C prior to seeding cells at the density of 14 x 10^3^ in 100 μl of endothelial basal medium (EBM) containing 2% of fetal bovine serum (FBS) and 10% of serum from lSSc or dSSc patients, naïve or under pharmacological therapy with CYC, or 10% of serum from healthy controls. Positive controls were obtained using complete EGM-2-MV medium, which contains vascular endothelial growth factor (VEGF) and 5% of FBS, to verify the efficiency of the assay (i.e., the capability of cells to form capillaries *in vitro*). Wells were photographed under a Nikon Eclipse T5100 inverted phase contrast microscope (Nikon, Tokyo, Japan) with a x4 objective at 24 hours after cell seeding in 4 predetermined spots/well. Branching points were independently counted by two independent observers in a blinded manner. The total number of branching points in the 4 photographic fields of each plate was considered indicative of the complexity of the capillary network formed. All experimental conditions were tested in duplicate.

### Wound healing assay

dMVECs were seeded into 12-multiwell plates at the density of 50 x 10^3^ cells/well in complete EGM-2-MV medium. Once at confluence, cells were starved in EBM with 2% FBS. After 2 hours, the medium was removed and the monolayer was scratched with a sterile 1000-μl pipette tip. The resulting wound was ~ 1 mm wide. After careful washing with phosphate-buffered saline (PBS), cells were fed with 1 ml of EBM containing 2% FBS and 10% of serum from lSSc or dSSc patients, naïve or under pharmacological therapy with CYC, or 10% of serum from healthy controls. Positive controls were obtained using complete EGM-2-MV medium to verify the efficiency of the assay. All experimental conditions were tested in duplicate. The wounded area was observed at 0, 6 and 24 hours after scratching. At 24 hours, a predetermined field encompassing almost all the wounded area was photographed under a Nikon Eclipse T5100 inverted microscope (Nikon) with a x10 objective. The percentage of repair was evaluated with the “Area fraction” function of the NIS-Elements software version 2.3 (Nikon).

### *In vitro* chemotaxis assay

Chemotaxis was assessed by using the Boyden chamber assay performed in 24-multiwell plates with inserts containing an 8-μm pore size polyethylene terephthalate (PET) membrane (BD Biosciences). The solution to be tested (750 μl of EBM containing 2% of FBS and 10% of serum from lSSc or dSSc patients, naïve or under pharmacological therapy with CYC, or 10% of serum from healthy controls) was placed in the lower chamber. Positive controls were obtained with complete EGM-2-MV medium to verify the efficiency of the assay. A chemokinetic effect was excluded using EBM in both the upper and the lower well. Under this condition we failed to detect any cell on the lower side of the membrane. A suspension of 25 x 10^3^ dMVECs/insert in EBM containing 2% of FBS was added in the upper chamber. All experimental conditions were tested in duplicate. At 24 hours after cell seeding the inserts with adhering cells were fixed *in situ* for 2 minutes with 3% formalin in PBS and then permeabilized for 20 minutes with methanol. Non-migrated cells were mechanically removed from the upper surface of the PET membrane by scrubbing with a cotton-tipped swab. Membranes were then stained for 15 minutes with Giemsa Stain (J.T. Baker; VWR International, Milan, Italy), washed with PBS, detached from the insert with a blade and mounted upside down on glass slides. Each membrane was photographed under a Nikon E600 light microscope (Nikon) with a x20 objective in 4 randomly selected fields. Migrated cells were counted in a blinded manner by two independent observers with the aid of the NIS-Elements software version 2.3 (Nikon).

### Cell proliferation assay

dMVECs were seeded into 96-multiwell plates (40 x 10^3^ cells/well) in complete EGM-2-MV medium and were left to adhere overnight. Cells were then washed 3 times with serum-free medium and incubated in EBM with 2% FBS for 24 hours. Subsequently, dMVECs were incubated for 24 hours in EBM containing 2% FBS and 10% of serum from lSSc or dSSc patients, naïve or under pharmacological therapy with CYC, or 10% of serum from healthy controls. The proliferative effect with complete EGM-2-MV medium was defined as the optimal growth and was set as 100% proliferation. Cell proliferation was determined by the Cell Proliferation Reagent WST-1 (4-[3-(4-iodophenyl)-2-(4-nitrophenyl)-2H-5-tetrazolio]-1,3-benzene disulfonate) colorimetric assay (Roche Diagnostics, Mannheim, Germany) according to the manufacturer’s instructions. All measurements were performed in triplicate.

### Detection of apoptosis by TUNEL assay

dMVECs were grown to confluence on glass coverslips, starved in EBM with 2% FBS overnight and then incubated for 24 hours in EBM containing 2% FBS and 10% of serum from lSSc or dSSc patients, naïve or under pharmacological therapy with CYC, or 10% of serum from healthy controls. dMVECs were subsequently fixed in 3.7% buffered paraformaldehyde and permeabilized with 0.1% Triton X-100 in PBS. For immunofluorescent detection and quantification of cell apoptosis we used the terminal deoxynucleotidyl transferase-mediated dUTP nick-end labeling (TUNEL) technology (Fluorescein Isothiocyanate (FITC) In Situ Cell Death Detection Kit; Roche Diagnostics) according to the manufacturer’s instructions. Nuclei were counterstained with 4’,6-diamidino-2-phenylindole (DAPI). The stained cells were observed under a Leica DM4000 B microscope (Leica Microsystems, Mannheim, Germany) and photographed using a Leica DFC310 FX 1.4-megapixel digital colour camera equipped with the Leica software application suite LAS V3.8 (Leica Microsystems). The percentage of apoptotic dMVEC nuclei was calculated as TUNEL/DAPI-positive nuclei in proportion to all DAPI-positive nuclei. Counting was performed on ten randomly chosen microscopic fields (x40 original magnification) per sample by two independent blinded observers.

### Enzyme-linked immunosorbent assay

Levels of the angiostatic mediators endostatin, pentraxin 3 (PTX3), angiostatin and matrix metalloproteinase-12 (MMP-12) in serum samples were measured by commercial quantitative colorimetric sandwich enzyme-linked immunosorbent assay (Human Endostatin Quantikine ELISA Kit and Human Pentraxin 3/TSG-14 Quantikine ELISA Kit, R&D Systems, Minneapolis, Minnesota, USA; Human Angiostatin ELISA Kit, RayBiotech, Norcross, Georgia, USA; Human Matrix Metallopeptidase 12 ELISA Kit, Antibodies-online, Atlanta, Georgia, USA) according to the manufacturer’s protocol. The detection range was 0.31–10 ng/ml for endostatin, 0.31–20 ng/ml for PTX3, 20–2000 ng/ml for angiostatin and 0.156–10 ng/ml for MMP-12. Serum samples were diluted 1:4 for the endostatin assay. Concentrations were calculated using a standard curve generated with specific standards provided by the manufacturer. Each sample was measured in duplicate.

### Statistical analysis

Data presented are means and standard errors of the mean (SEM). Statistical analysis was performed using the Student’s *t*-test for independent groups. A p-value less than 0.05 according to a two-tailed distribution was considered statistically significant.

## Results

### Demographic and clinical data of SSc patients

The demographic, clinical and serological characteristics of the SSc patients enrolled in the study are listed in Table 1. As reported in Table 1, CYC was given for 12–18 months to thirteen patients (three males and ten females), in eight cases for interstitial lung disease and in the remaining five cases for severe and rapidly progressive cutaneous involvement during the early phase of the disease; six of them were anti-Scl70 positive.

**Table 1:** Demographic and clinical characteristics of patients with SSc.

Age, mean ± SD (years)	56.5 ± 12.1
Male	4 (19.1%)
Female	17 (80.9%)
lSSc subset	13 (61.9%)
dSSc subset	8 (38.1%)
Disease duration, mean ± SD (years)^§^	7.7 ± 4.1
ANA	21 (100%)
Anti-Scl70	7 (33.3%)
ACA	10 (47.6%)
Digital ulcers	12 (57.1%)
Early NVC pattern	4 (19.1%)
Active NVC pattern	8 (38.1%)
Late NVC pattern	9 (42.9%)
Skin score, mean ± SD	10.2 ± 6.3
ILD^#^	11 (52.4%)
CYC treatment	13 (61.9%)^¥^

ACA, anticentromere antibodies; ANA, antinuclear antibodies; Anti-Scl70, anti-Scl70 antibodies; CYC, cyclophosphamide; dSSc, diffuse cutaneous systemic sclerosis; ILD, interstitial lung disease; lSSc, limited cutaneous systemic sclerosis; NVC, nailfold videocapillaroscopy; SSc, systemic sclerosis.

Except where indicated otherwise, values are the absolute number and percentage of patients.

^**§**^Disease duration was calculated since the first non-Raynaud’s symptom of SSc.

^#^Determined by high-resolution computed tomography scan.

^¥^Eight patients with lSSc and five patients with dSSc.

### Effects of SSc sera on *in vitro* capillary morphogenesis

**Fig 1:**
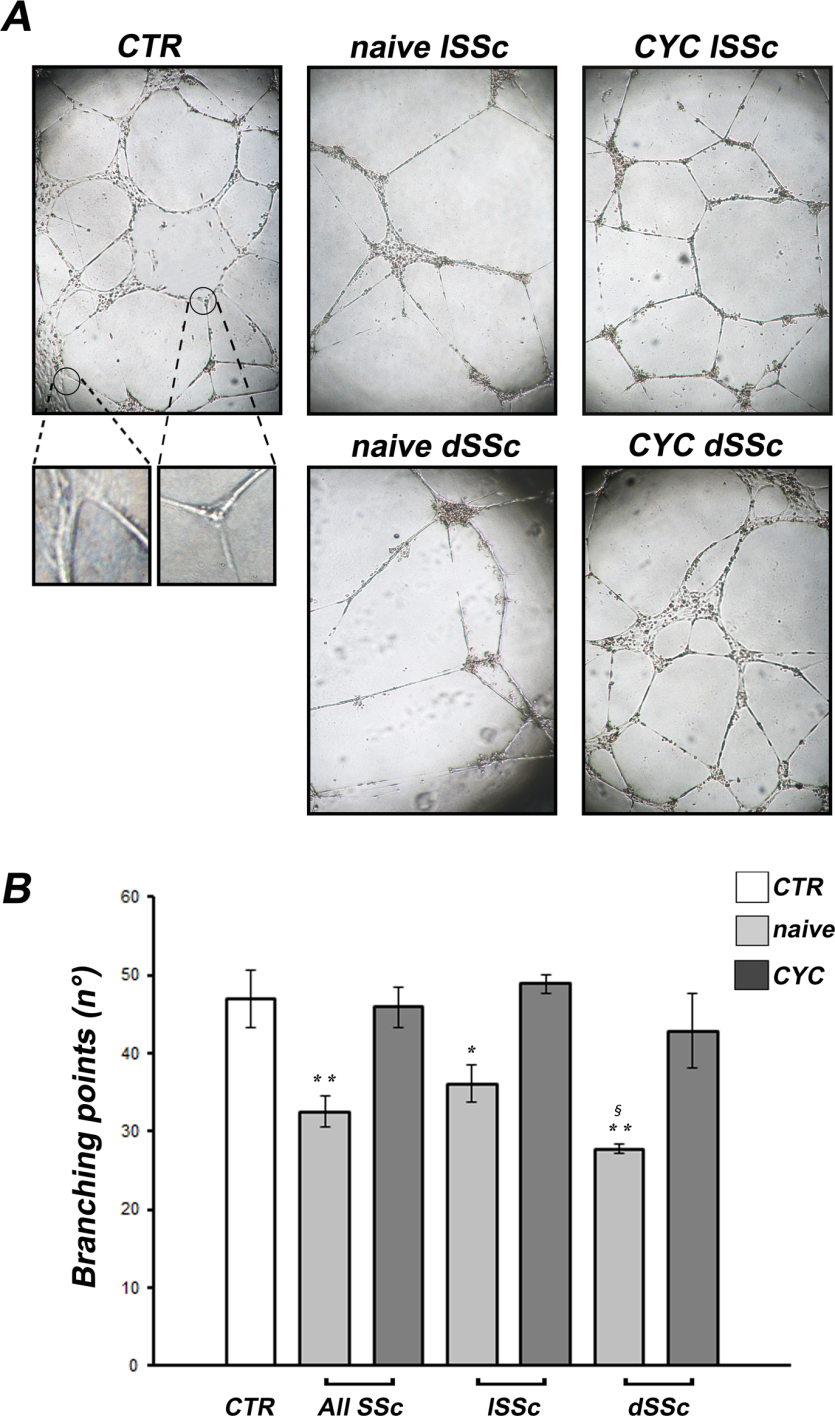
Systemic sclerosis (SSc) sera impair the ability of dermal microvascular endothelial cells (dMVECs) to form capillary-like tubes on Geltrex matrix. *In vitro* capillary morphogenesis of dMVECs was evaluated after challenge with sera from healthy controls (CTR, n = 8) and SSc patients, treatment-naïve (n = 8) or under cyclophosphamide (CYC) treatment (n = 13). **(A)** Representative images of the capillary network formed on Geltrex at 24 hours from plating are shown for each experimental point. Original magnification, ×4. The square panels at the bottom provide higher magnification views of the encircled areas in CTR panel to demonstrate the presence of capillary-like structures provided with a lumen. **(B)** Quantitative analysis of the number of branching points as a measure of the complexity of the capillary network formed. Data are means ± SEM of three independent experiments performed in duplicate with each one of the three dMVEC lines. *p<0.05 and **p<0.005 vs. healthy controls, ^§^p = 0.02 vs. naïve lSSc. dSSc, diffuse cutaneous SSc; lSSc, limited cutaneous SSc.

dMVECs formed capillary-like tubes in all experimental conditions assayed (Fig 1A). At 3 hours after seeding on Geltrex, cells had already aligned and formed linear capillary-like structures. After 6–8 hours, new branches sprouted from the pre-existing ones, and at 24 hours a network of closed capillary-like structures provided with a lumen had formed (Fig 1A).

The number of branching points was significantly lower upon challenge of dMVECs with sera from treatment-naïve SSc patients compared with healthy controls (p<0.005) (Fig 1B). In particular, although to a different extent, differences between either naïve lSSc or naïve dSSc sera and healthy control sera were statistically significant (p<0.05 and p<0.005, respectively) (Fig 1B). Moreover, the angiogenic response was significantly lower in the presence of naïve dSSc sera compared with naïve lSSc sera (p = 0.02) (Fig 1B). When dMVECs were challenged with sera from CYC-treated lSSc and dSSc patients, their angiogenic response was comparable to that of cells treated with healthy sera (Fig 1B). Accordingly, either when considering the whole SSc group or the lSSc and dSSc subsets separately, the angiogenic performance of dMVECs was significantly greater upon challenge with sera from CYC-treated patients compared with naïve patients (p<0.005 for all comparisons).

### Effects of SSc sera on wound healing capacity of dMVECs

**Fig 2:**
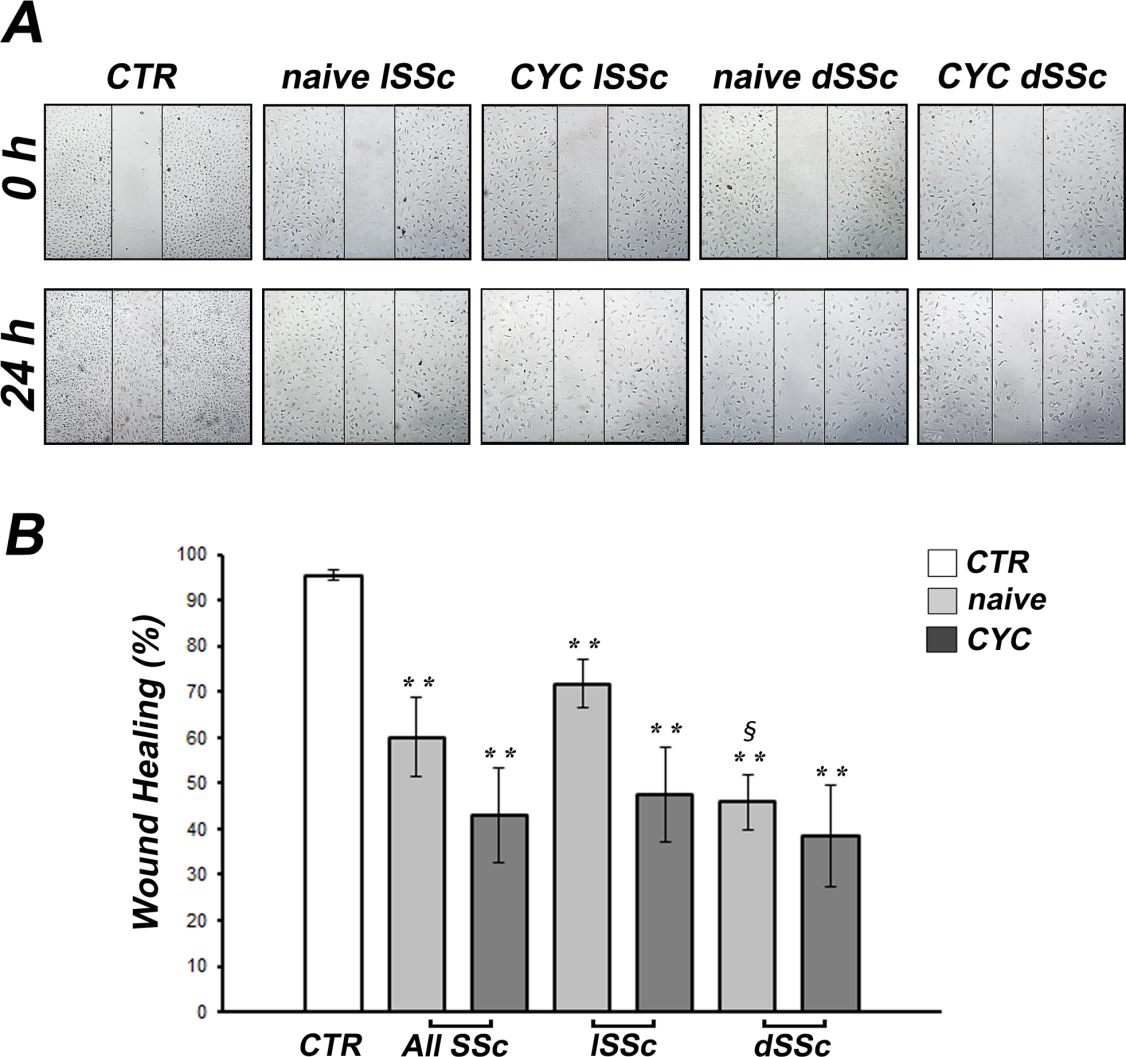
Systemic sclerosis (SSc) sera impair wound healing capacity of dermal microvascular endothelial cells (dMVECs). Wound healing capacity of dMVECs was assayed in the presence of sera from healthy controls (CTR, n = 8) and SSc patients, treatment-naïve (n = 8) or under cyclophosphamide (CYC) treatment (n = 13). **(A)** Representative images of the wounded area at 0 hours and 24 hours after scratching. Original magnification, ×10. **(B)** Quantitative analysis of the percentage of wound repair. Data are means ± SEM of three independent experiments performed in duplicate with each one of the three dMVEC lines. **p<0.005 vs. healthy controls, ^§^p<0.001 vs. naïve lSSc. dSSc, diffuse cutaneous SSc; lSSc, limited cutaneous SSc.

After scratching in the presence of healthy control sera, dMVECs migrated into the wounded area and then proliferated, and at 24 hours the monolayer integrity was completely restored (Fig 2A). Conversely, at 24 hours after scratching in the presence of sera from both lSSc and dSSc patients, dMVECs were unable to restore the monolayer integrity (Fig 2A). Indeed, wound healing capacity was significantly decreased upon challenge of dMVECs with sera from both treatment-naïve and CYC-treated SSc patients compared with healthy controls (both p<0.005), without any significant difference between naïve and CYC-treated SSc sera (Fig 2B). Furthermore, wound healing capacity was significantly lower when dMVECs were challenged with naïve dSSc sera compared with naïve lSSc sera (p<0.001) (Fig 2B).

### Effects of SSc sera on *in vitro* chemotaxis of dMVECs

**Fig 3:**
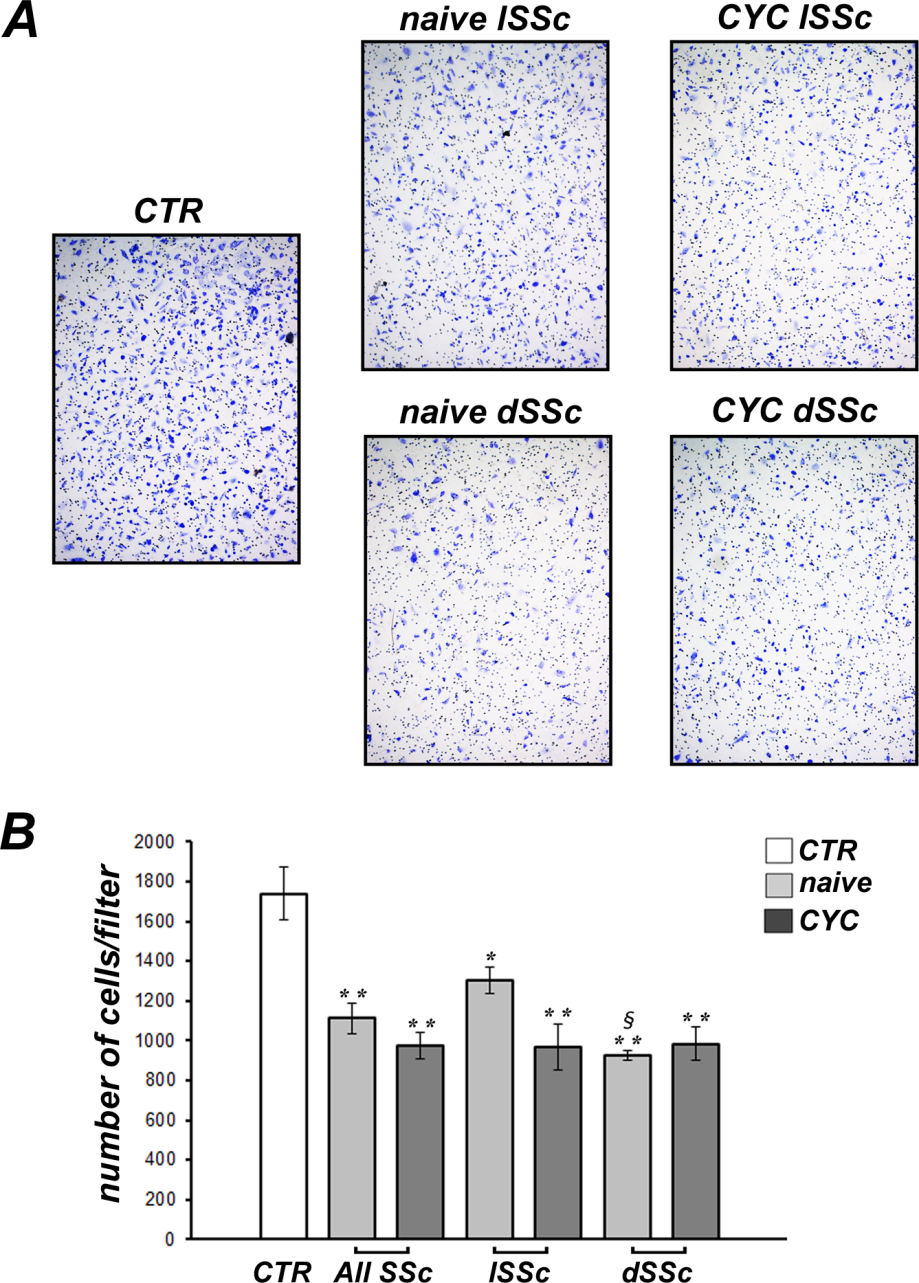
Systemic sclerosis (SSc) sera impair chemotaxis of dermal microvascular endothelial cells (dMVECs). Chemotaxis of dMVECs was assessed by using the Boyden chamber assay, placing in the lower compartment sera from healthy controls (CTR, n = 8) and SSc patients, treatment-naïve (n = 8) or under cyclophosphamide (CYC) treatment (n = 13). **(A)** Representative images of the filters showing migrated cells labeled with Giemsa Stain. Original magnification, ×4. **(B)** Quantitative analysis of chemotaxis expressed as the number of migrated cells per filter. Data are means ± SEM of three independent experiments performed in duplicate with each one of the three dMVEC lines. *p<0.05 and **p<0.001 vs. healthy controls, ^§^p<0.001 vs. naïve lSSc. dSSc, diffuse cutaneous SSc; lSSc, limited cutaneous SSc.

The chemotaxis assay revealed a prominent inhibitory effect of sera from both lSSc and dSSc patients on dMVEC migration (Fig 3A). Indeed, dMVECs challenged with either treatment-naïve SSc or CYC-treated SSc sera displayed significantly lower chemotactic capacity compared with cells challenged with healthy sera (both p<0.001), with no significant difference between naïve and CYC-treated SSc sera (Fig 3B). Moreover, chemotaxis was significantly lower in the presence of naïve dSSc sera compared with naïve lSSc sera (p<0.001) (Fig 3B).

### Effects of SSc sera on dMVEC proliferation and apoptosis

**Fig 4:**
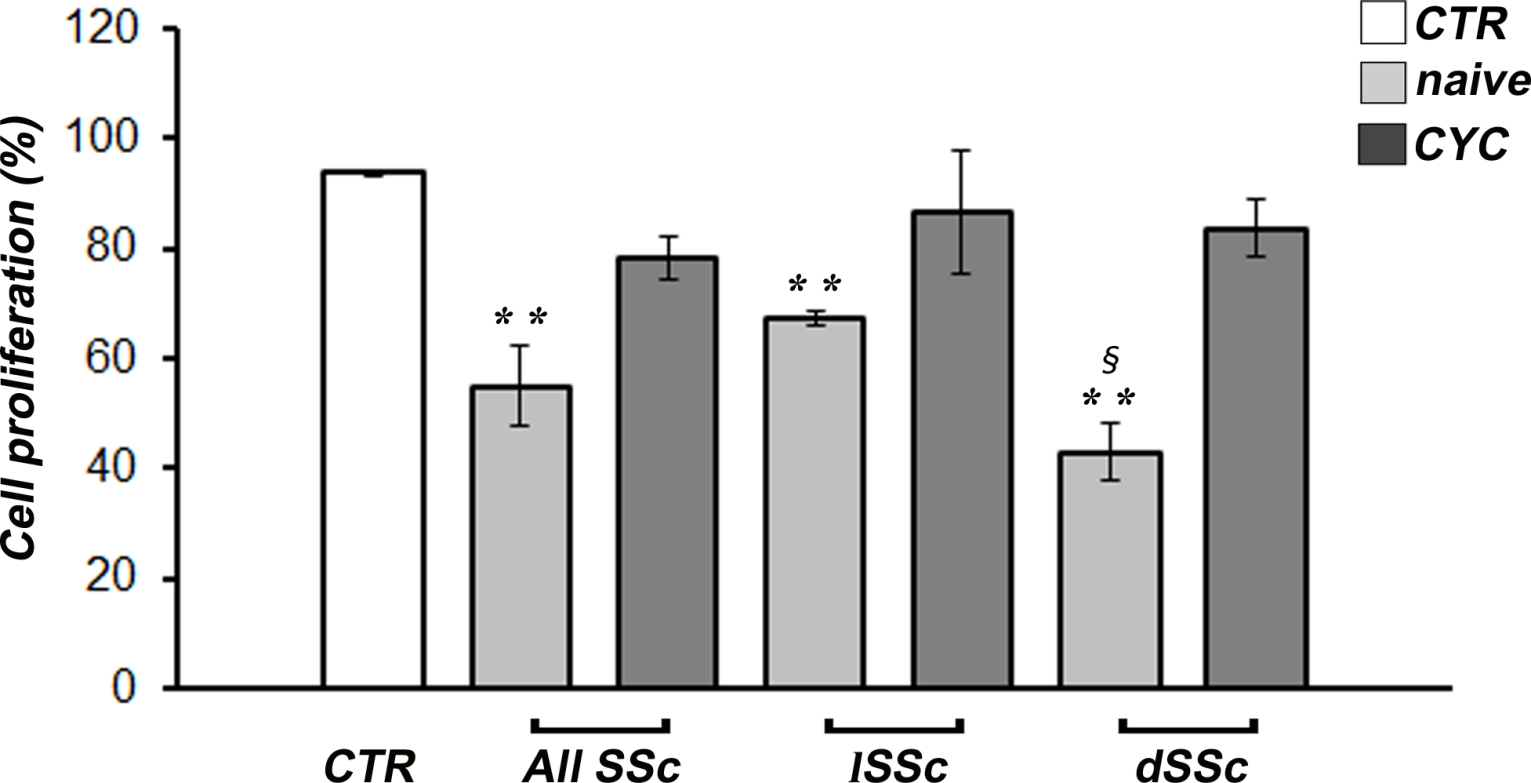
Systemic sclerosis (SSc) sera impair proliferation of dermal microvascular endothelial cells (dMVECs). Cell proliferation was measured by the WST-1 colorimetric assay after challenge for 24 hours with sera from healthy controls (CTR, n = 8) and SSc patients, treatment-naïve (n = 8) or under cyclophosphamide (CYC) treatment (n = 13). Cell proliferation in the presence of complete EGM-2-MV medium was set to 100%; all results are normalized to this value. Data are means ± SEM of three independent experiments performed in duplicate with each one of the three dMVEC lines. **p<0.01 vs. healthy controls. dSSc, diffuse cutaneous SSc; lSSc, limited cutaneous SSc.

Either when considering the whole SSc group or the lSSc and dSSc subsets separately, cell proliferation was significantly decreased in dMVECs challenged with sera from naïve SSc patients compared with healthy sera (p<0.01 for all comparisons) (Fig 4). Moreover, cell proliferation was significantly lower in the presence of naïve dSSc sera compared with naïve lSSc sera (p = 0.01) (Fig 4). Conversely, when dMVECs were challenged with sera from CYC-treated lSSc and dSSc patients, cell proliferation was comparable to that of cells treated with healthy sera (Fig 4).

**Fig 5:**
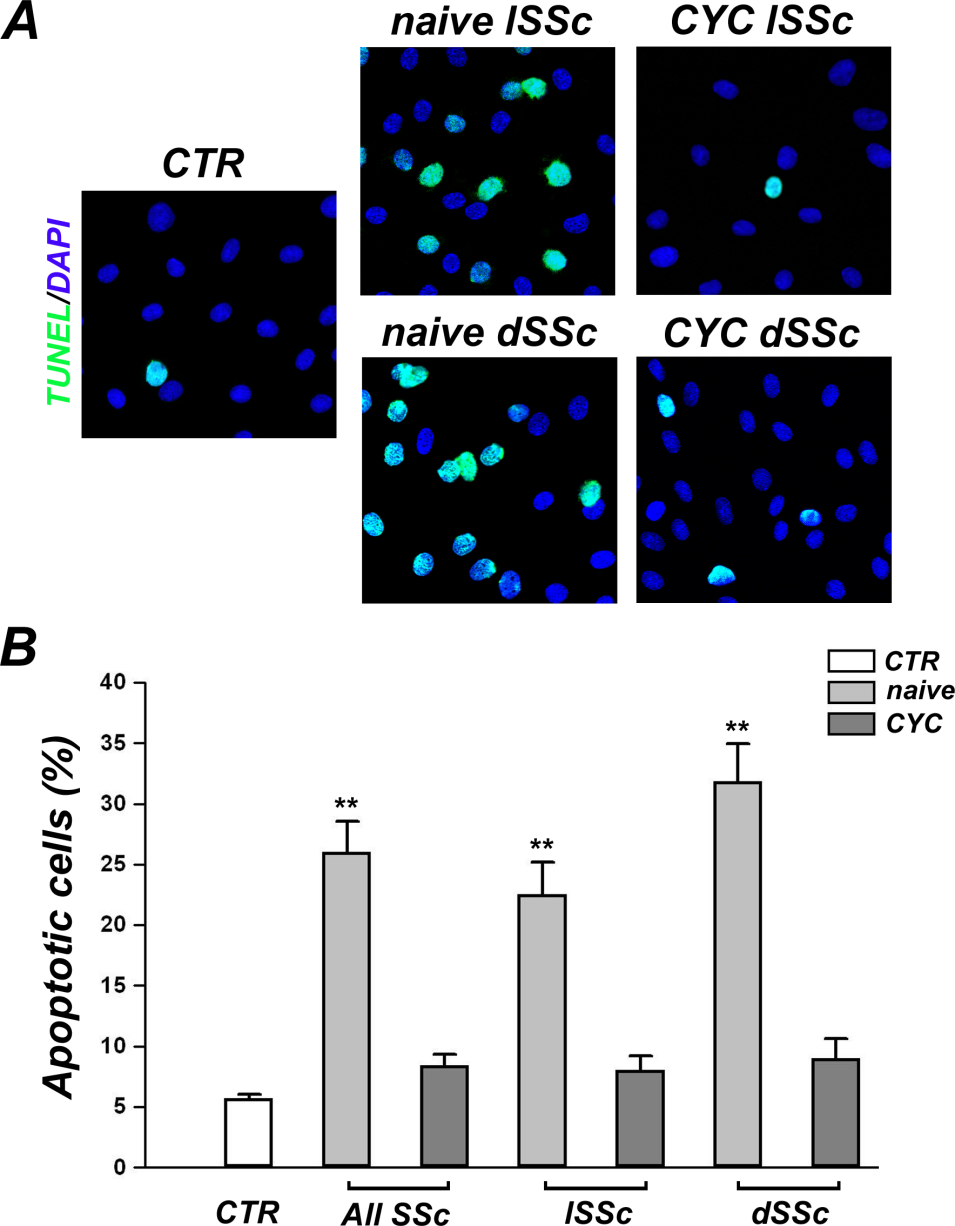
Systemic sclerosis (SSc) sera induce apoptosis of dermal microvascular endothelial cells (dMVECs). Apoptosis was evaluated by fluorescent TUNEL assay after challenge for 24 hours with sera from healthy controls (CTR, n = 8) and SSc patients, treatment-naïve (n = 8) or under cyclophosphamide (CYC) treatment (n = 13). **(A)** Representative images of dMVECs subjected to FITC-TUNEL (green) labeling and DAPI (blue) counterstaining for nuclei. Apoptotic nuclei are green/blue double stained. Original magnification, ×20. **(B)** Quantitative analysis of the percentage of apoptotic dMVECs. Data are means ± SEM of three independent experiments performed in duplicate with each one of the three dMVEC lines. **p<0.001 vs. healthy controls. dSSc, diffuse cutaneous SSc; lSSc, limited cutaneous SSc.

dMVEC apoptosis in the different experimental conditions was assessed by fluorescent TUNEL assay (Fig 5A). The percentage of apoptotic cells was significantly increased upon challenge of dMVECs with sera from naïve SSc patients compared with healthy controls (p<0.001) (Fig 5B). In particular, differences between either naïve lSSc or naïve dSSc sera and healthy sera were statistically significant (both p<0.001) (Fig 5B). Furthermore, apoptosis was greater in the presence of naïve dSSc sera than naïve lSSc sera, but this difference did not reach statistical significance (p = 0.07) (Fig 5B). Of note, no difference in apoptosis rate was observed between dMVECs challenged with sera from CYC-treated SSc patients and those treated with healthy sera (Fig 5B). Consistent with these findings, the percentage of apoptotic dMVECs was significantly lower upon challenge with sera from CYC-treated patients compared with sera from naïve patients (p<0.001).

### Levels of angiostatic factors in naïve and CYC-treated SSc sera

**Fig 6:**
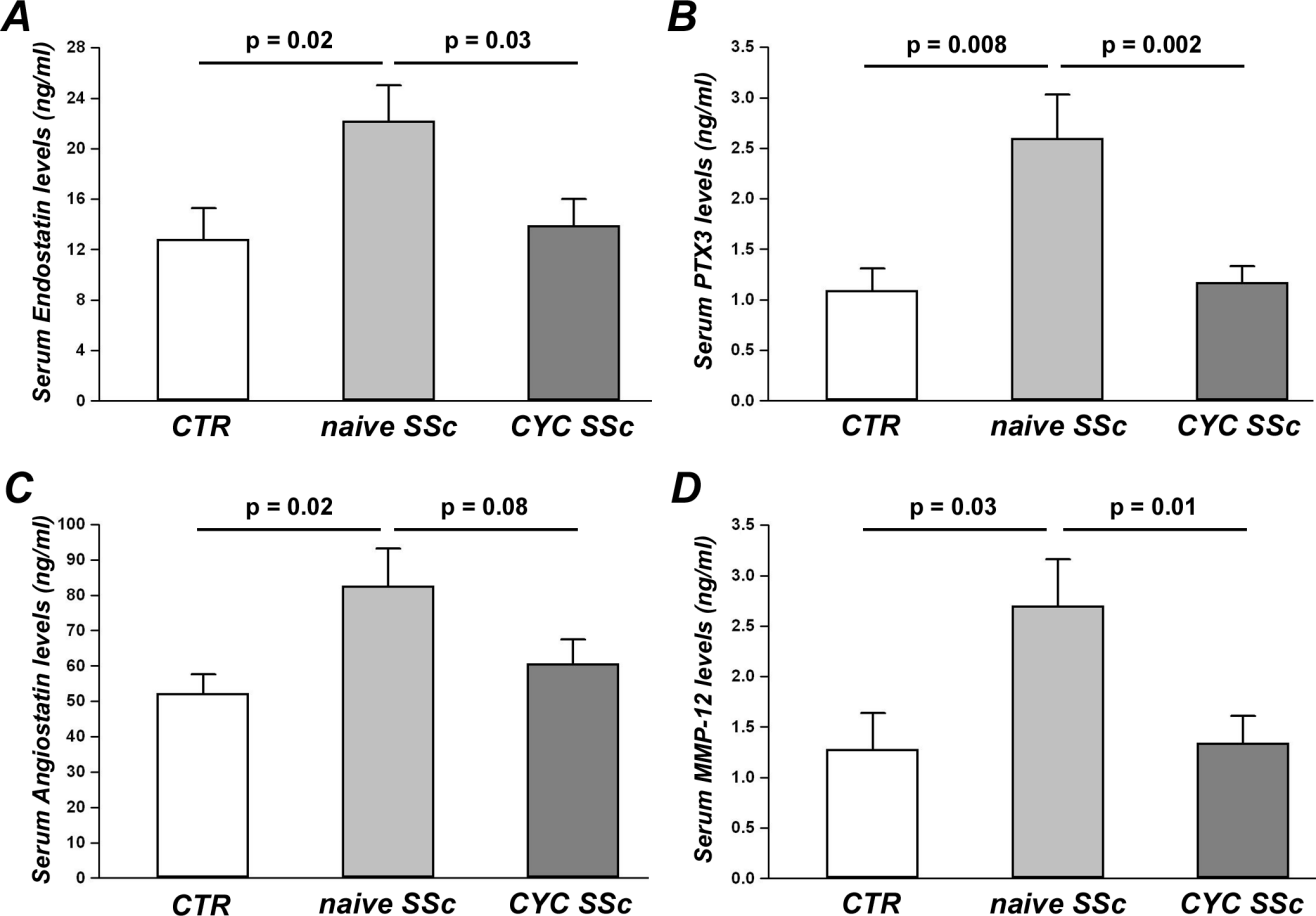
Serum levels of negative angiogenesis regulators. Levels of endostatin **(A)**, pentraxin 3 (PTX3) **(B)**, angiostatin **(C)** and matrix metalloproteinase-12 (MMP-12) **(D)** were measured by quantitative colorimetric sandwich ELISA in sera from healthy controls (CTR, n = 8) and SSc patients, treatment-naïve (n = 8) or under cyclophosphamide (CYC) treatment (n = 13). Data are means ± SEM. p-values are indicated in each panel.

Serum levels of endostatin were significantly increased in naïve SSc patients (22.21 ± 2.80 ng/ml) compared both with healthy controls (12.84 ± 2.52 ng/ml, p = 0.02) and CYC-treated SSc patients (13.95 ± 2.11 ng/ml, p = 0.03) (Fig 6A). As displayed in Fig 6B, levels of PTX3 in naïve SSc sera (2.60 ± 0.43 ng/ml) were significantly higher than in control sera (1.09 ± 0.22 ng/ml, p = 0.008) and CYC-treated SSc sera (1.17 ± 0.16 ng/ml, p = 0.002). Serum angiostatin was significantly increased in naïve SSc patients (82.59 ± 10.57 ng/ml) compared to controls (52.19 ± 5.49 ng/ml, p = 0.02) (Fig 6C). Moreover, angiostatin was higher in naïve SSc than in CYC-treated SSc sera (60.65 ± 6.82 ng/ml), although this difference was not statistically significant (p = 0.08) (Fig 6C). A significant increase in serum levels of MMP-12 was detected in naïve SSc patients (2.70 ± 0.46 ng/ml) compared with controls (1.28 ± 0.36 ng/ml, p = 0.03) and CYC-treated SSc patients (1.34 ± 0.27 ng/ml, p = 0.01) (Fig 6D). No difference in serum levels of endostatin, PTX3, angiostatin and MMP-12 was found between healthy controls and CYC-treated SSc patients (Fig 6A–6D).

## Discussion

Our study demonstrates that i) *in vitro* angiogenesis and survival of dMVECs are significantly and differentially impaired by sera from lSSc and dSSc patients, these latter displaying the strongest inhibitory effect, and ii) treatment with the powerful immunosuppressant CYC may effectively abolish such antiangiogenic and proapoptotic actions, possibly explaining the CYC-mediated improvement of SSc peripheral microangiopathy observed in clinical practice [32].

The different *in vitro* assays used in this work exploit different endothelial cell processes required during angiogenesis. The capillary tube assay on Geltrex allows to evaluate the ability of MVECs to organize in a physiologic extracellular matrix. Geltrex is in fact a soluble form of reduced growth factor basement membrane extract purified from murine Engelbreth-Holm-Swarm tumor and, like Matrigel, is rich in laminin, collagen IV, entactin, and heparan sulfate proteoglycan. Upon seeding on Geltrex, MVECs rapidly form capillary-like tubes. Since during *in vitro* angiogenesis endothelial cells first align and then sprout from the neoformed capillary vessels to form a network, we quantitatively assayed the angiogenic process by counting the number of branching points as a direct measure of the complexity of the mesh formed.

To recapitulate *in vitro* a physiologic or pathologic bloodstream environment, we exposed dMVECs to sera from healthy controls or SSc patients, which were treatment-naïve or under CYC treatment. In the presence of sera from both naïve lSSc and dSSc patients, the number of branching points formed by dMVECs was significantly lower compared to cells challenged with healthy sera. These findings are in agreement with a previous work demonstrating antiangiogenic plasma activity in patients with SSc, which was mainly attributed to the increased levels of antiangiogenic angiostatin [36]. Moreover, here we report for the first time that the antiangiogenic activity of dSSc sera is significantly greater than that of lSSc sera, which is consistent with the higher prevalence of most severe peripheral vascular manifestations reported in the dSSc subset, such as avascular areas on nailfold videocapillaroscopy and digital ulcers [37–39]. This reduced capacity to form capillary-like tubes *in vitro* is likely to be imputed to the profound imbalance between proangiogenic and angiostatic factors described in the SSc bloodstream [5,10–16,40,41]. Indeed, we herein show that our naïve SSc sera contain increased levels of different negative angiogenesis regulators, including endostatin, angiostatin, PTX3 and MMP-12. Endostatin and angiostatin are considered among the most powerful angiogenesis inhibitors [10,36]. PTX3 is a multifunctional pattern recognition protein that can suppress the proangiogenic action of fibroblast growth factor 2 (FGF2) and has been associated with SSc vascular manifestations and defective angiogenesis [20,42]. In SSc, elevated levels of MMP-12 may suppress angiogenesis through the cleavage and subsequent inactivation of endothelial urokinase-type plasminogen activator receptor, as well as through the proteolysis of plasminogen and generation of angiostatin [15,19,20]. In addition, SSc serum has been reported to contain anti-endothelial cell antibodies that may induce endothelial cell apoptosis [43–45] and anti-fibrillin-1 antibodies that may alter the interaction of endothelial cells with the surrounding extracellular matrix [46,47]. In agreement with these findings, we also observed an increased apoptosis rate in dMVECs upon incubation with naïve SSc sera.

Of note, such antiangiogenic and proapoptotic effects were observed by using sera from both lSSc and dSSc patients who were not under treatment with immunosuppressant or disease-modifying drugs. Conversely, the addition of sera from CYC-treated SSc patients did not impair the angiogenic ability of dMVECs, as shown by a number of branching points comparable to that of cells challenged with healthy sera. Thus, CYC treatment seems to be able to block the antiangiogenic activity of SSc sera. CYC is a potent immunosuppressant used in the treatment of different autoimmune diseases such as systemic lupus erythematosus, vasculitis, myositis and also SSc, especially in patients with interstitial lung disease [48,49]. As the last CYC infusion has been administered to our patients 1 month before blood withdrawal, this beneficial action on angiogenesis is unlikely to be a direct effect of the drug. CYC treatment might rather have rebalanced circulating levels of positive and negative regulators of angiogenesis, possibly *via* its immunosuppressive action. Indeed, CYC exerts its anti-inflammatory and immunosuppressive functions through direct cytotoxicity on bone marrow precursors and mature immune cells, mainly B and T cells [50]. During angiogenesis, cells of both the innate and adaptive immune systems are involved in the mechanisms of endothelial cell activation, proliferation and migration through the production and release of a large spectrum of angiogenic mediators [51]. In this regard, *in vitro* studies on peripheral blood mononuclear cells suggested a defective contribution of SSc lymphocytes and monocytes to angiogenesis [52,53]. Furthermore, supernatants from SSc peripheral blood mononuclear cells decreased endothelial cell chemotaxis [54], and SSc serum failed to enhance normal mononuclear cell angiogenic capacity [55]. Therefore, our findings might be explained by the possible CYC-mediated modulation of immune cell-derived proangiogenic and antiangiogenic molecules. To explore this hypothesis, we looked at possible differences in serum levels of some antiangiogenic factors that have been implicated in SSc [10,15,19,20,36,42]. Strikingly, we observed significantly reduced levels of endostatin, angiostatin, PTX3 and MMP-12 in CYC-treated SSc sera compared with naïve SSc sera. Indeed, circulating levels of the aforementioned molecules in CYC-treated SSc patients were comparable to healthy controls. In addition, CYC treatment might have reduced serum levels of autoantibodies acting on the endothelium, including proapoptotic anti-endothelial cell antibodies and anti-fibrillin-1 antibodies, *via* depletion of autoreactive B lymphocytes. In this regard, we found that the percentage of TUNEL-positive apoptotic dMVECs was significantly higher in the presence of sera from naïve SSc patients compared with healthy controls, while CYC-treated SSc sera did not induce dMVEC apoptosis. However, at this stage we did not investigate possible differences in the presence of such autoantibodies between naïve and CYC-treated SSc sera.

In an experimental setting quite similar to the present one, we have previously shown that stimulation of dMVECs with CYC-treated SSc sera preserved their ability to synthesize fibrillin-1 and the expression of the adhesion molecule α_v_β_3_ integrin, which instead were both impaired on challenge with naïve SSc sera [33]. Of note, α_v_β_3_ integrin is a crucial angiogenesis regulator [56] and fibrillin-1 has been reported to induce α_v_β_3_ integrin-mediated signaling in human endothelial cells [57]. Thus, CYC treatment might exert a proangiogenic effect by maintaining endothelial α_v_β_3_ integrin expression and fibrillin-1 deposition at normal levels. Nevertheless, further in-depth studies will help to provide a mechanistic explanation for the differential serum effects reported herein. In addition to its possible impact on fully differentiated endothelial cell functions, it is noteworthy that in SSc patients CYC treatment may even promote the mobilization of bone marrow-derived endothelial progenitor cells involved in vasculogenesis [58].

In accordance with the reduced angiogenesis observed on Geltrex, both dMVEC chemotaxis and wound healing capacity were also greatly reduced in all the cultures challenged with naïve SSc sera, with the strongest inhibitory effects being observed in the presence of dSSc sera. The Boyden chamber assay exploits the capacity of cells to migrate in response to a chemoattractant placed in the lower chamber, in our case SSc or healthy serum. The reduced cell migration observed in the presence of sera from naïve SSc patients may suggest the action of some chemotaxis inhibitors. As an example, endostatin, which is increased in the serum of SSc patients [10,40,59], has been shown to inhibit VEGF-induced human umbilical vein endothelial cell migration [60]. Other factors or autoantibodies present in SSc bloodstream might interfere with the main promoters of endothelial cell migration, including FGF2, angiopoietins and platelet-derived growth factor [61]. At variance with the Boyden chamber assay, the wound healing assay does not require only migration of cells but also proliferation, which may have been impaired by transforming growth factor-β (TGF-β) or other cytotoxic factors present in the serum of SSc patients. Indeed, TGF-β has been shown to inhibit cell proliferation and induce apoptosis of endothelial cells with a variety of mechanisms [62–64]. Interestingly, unlike what we observed in the *in vitro* capillary morphogenesis assay, we could not find any significant difference between the effects of naïve and CYC-treated SSc sera either on dMVEC chemotaxis or wound healing capacity. Of note, we further specifically assessed the effects of SSc sera on endothelial cell proliferation using the WST-1 assay. Our findings revealed that cell proliferation was significantly decreased in dMVECs challenged with sera from naïve SSc patients compared with healthy sera. Conversely, dMVEC proliferation was preserved in the presence of sera from CYC-treated SSc patients. Thus, the lack of wound healing capacity observed in the presence of CYC-treated SSc sera is likely to be attributed to a selective impairment of dMVEC migration. Taken together, CYC treatment seems unable to specifically counteract the anti-migratory effects of SSc sera. However, we should also consider that because of the lack of a matrix substrate, chemotaxis and wound healing assays do not adequately reflect *in vitro* what is happening *in vivo*. Instead, as assessed by capillary morphogenesis on Geltrex matrix, it is noteworthy that even in the presence of CYC-treated SSc sera dMVECs could sustain a normal angiogenic program in a microenvironment which better reflects the *in vivo* situation. This might be related to a rebalance of abnormalities in endothelial cell-matrix interactions following CYC treatment, as suggested by our previous work [33].

## Conclusions

In summary, our data clearly show that sera from naïve SSc patients significantly impair *in vitro* capillary morphogenesis, wound healing capacity, chemotaxis and survival of dMVECs. Among the endothelial cell functions investigated herein, cell proliferation and the ability to organize in capillary-like tubes were maintained at normal levels following CYC treatment. In SSc, CYC treatment might boost angiogenesis and consequently improve peripheral microangiopathy mainly through the normalization of the endothelial cell-matrix interactions, reduction of endothelial cell apoptosis and rebalance of dysregulated angiostatic factors.
